# The Homœopathic Medical Society of Indiana

**Published:** 1894-09

**Authors:** 


### THE HOMŒOPATHIC MEDICAL SOCIETY OF INDIANA.

The twenty-eighth annual meeting convened in the parlors of the Grand Hotel, Indianapolis, Thursday, May 17th, 1894. President M. H. Waters, of Terre Haute, was in the chair, and the attendance from all parts of the State was good. Several noted men from other States were present, among them Dr.

J. D. Buck, President of the Pulte Medical College, Cincinnati, and Dr. C. E. Walton, Professor of Surgery in that college; Dr. I. L. Dryden, of St. Louis; Dr. H. B. Fellows, Professor of Mental and Nervous Diseases in Hahnemann Medical College, Chicago. These gentlemen, by invitation, addressed the Institute.

After the report of the Treasurer, Dr. J. S. Martin, of Muncie, showing the finances to be in a healthy state, and the report of the Secretary, Dr. W. B. Clarke, of Indianapolis, the President's address was delivered by Dr. Waters. This showed the progress in medicine since the last meeting, and dealt somewhat with the germ theory. He spoke of the homœopathic school as the only one to study drugs by their action on the healthy body for the purpose of guiding the physician to their use as remedies in cases of sickness. All of the members of the Institute must be students, ever on the alert for new facts. The disciples of Hahnemann had been ridiculed and misrepresented, and even now they had not succeeded in gaining positions in any of the public institutions. It was owing to the study by homœopaths that the origin of zymotic diseases had been determined, and it had been demonstrated that bacilli were the cause of disease. The knowledge so obtained had enabled the medical profession to control infectious diseases and stamp cholera and typhoid fever out of existence. He hoped that before long consumption would be recognized as an infectious disease, so that persons would guard against the germs as they do now against the germs of small-pox. He said that members of the Institute must watch their legal rights. Homœopaths were alone to blame for the lack of recognition accorded them. They did not push themselves enough. In conclusion, he advised the publishing of a journal of the proceedings of the annual meeting, so that the papers read could be preserved. He said that the physician of the future would be the guardian of the health of the people, and would not only help people to overcome sickness, but would teach them how to live so as to retain their health.

After a number of valuable papers had been read, Dr. William B. Clarke, of Indianapolis, noted for his many valuable contributions to the medical journals, and for his popular articles of a highly instructive character in the secular press, read a paper, entitled,

"VACCINATION NO SAFEGUARD."

"Ever since Jenner so cunningly grasped the grotesque common-folk cow-pox superstition and foisted it upon a credulous medical world with the bombastic and utterly unauthorized and unproved assertion, 'Whoever is once vaccinated with cow-pox is forever protected from small-pox,' has the warfare waged as to whether the proposition is true or untrue. A hundred years ought to be sufficient to prove either its truth or falsity, but although it has never been proved true there are people who still believe in it. Certain it is, however, that the opposition to its truth has crystallized into such a solid mass of evidence that no one can inspect it and retain a belief in the prophylactic efficacy of vaccination.

"No one who knows anything about the subject of vaccination will now assert that vaccination is protective very long, but many profess that frequent vaccinations are effective. Revaccination, then, is now on trial, vaccination being a confessed failure; but many professional men are now ready to kick the whole thing, vaccination, revaccination, and modification, overboard and depend as they should on sanitation, isolation, and treatment.

"Because a man was vaccinated and did not take the small-pox when exposed to it is no proof of the efficacy of vaccination, but the fact that millions who have been vaccinated did take the disease is proof of its inefficacy. The man who did not take it was protected by his own good health and the ability of his system to repel contagion. Hundreds of thousands of persons in Europe, in 1871, who had been vaccinated, took the disease, and again in India, in 1884. Out of nine hundred and seventy-four cases of small-pox in Bradford, England, last year seven hundred and two had been vaccinated. Out of five thousand cases in Philadelphia at least three thousand five hundred had been vaccinated. These statistics were made by the friends of vaccination, and it would be safe to say that all doubtful cases were placed in the not vaccinated list."

The doctor produced some strong evidence in support of the anti-vaccination theory, beginning with the experience of Jenner and his enthusiastic followers and coming down to the present day. He said it was surprising that, in the face of so much proof against the value of vaccination, the practice should still prevail. The subject had been periodically investigated by the greatest medical students of the world, and in nearly every instance the decision was against the practice. Often the investigation was made by strong friends of vaccination, and it was with great reluctance they changed their views. Dr. A. Vogt, professor of hygiene and sanitary statistics at Berne, collected the particulars of four hundred thousand cases of small-pox, and was finally compelled to admit that his "belief in vaccination was absolutely destroyed." Chauveau, in a paper before the French Academy of Medicine, in 1891, after detailing his experiments, concludes: "Vaccine virus never gives small-pox to man; variolic virus never gives vaccinia to the cow; vaccine is not even attenuated small-pox."

"How then," asks Dr. Clarke, "there being no resemblance between the two, can it be believed that vaccine virus can confer upon man immunity from small-pox? In these Pasteur and Koch days of bacteriological culture and of the germ theory craze of belief, is it not passing strange that the patient investigators along these lines are not paying some attention to the small-pox pustule itself as the source of vaccinating material for the possible prevention of small-pox, rather continuing to rely on pus from the cow (possibly a tuberculous cow), which possesses nothing in common with small-pox and which has so often been proved worthless?"

The doctor showed that vaccination was the cause of numerous diseases of the blood and skin, and was the cause of much suffering and death. It was the cause of many persons taking small-pox, he said, rather than of preventing them taking it, for he argued that during a scourge of the disease persons are vaccinated, and thus their systems are weakened and less able to repel the disease, and they fall victims to it. In conclusion, he said: "Vaccination is evil in its principles, false in its reasons, deadly in its results, and its compulsory enforcement is a desecration of sacred and ancient human rights, a breaker of homes, and a persecution of the poor. It is the largest sanitary question of the age, and if physicians do not settle it soon, it will be out of their hands and in those of the people, where it really belongs."

A paper on the "Progress of Bacteriology" was read by Dr. Theodore Potter, of Indianapolis. In conclusion, he said:

"In a former report I endeavored to summarize briefly the status of our knowledge as to the relationship between particular diseases and particular bacteria. First, tuberculosis: It has been demonstrated that tubercle bacilli may long remain latent or dormant in the body, to be called into activity by various exciting agencies. Thus the bronchial glands have been shown to contain living tubercle bacilli, while the lungs and other organs were apparently free both from the germs and the disease. This fact doubtless explains many cases of acute tuberculosis following such diseases as measles, whooping cough, and typhoid fever and slight wounds to bones and joints. Proof positive has been accumulating that actual congenital transmission of tuberculosis is more frequent than was a few years ago supposed. The question of compulsory notification of tuberculosis has given rise to sharp debate, but of the necessity of some measures to restrict the reckless spread of the disease there can be no difference of opinion.

"Erysipelas: It remains uncertain whether there is a specific germ of this disease or not.

"Tetanus: There seems to be no longer any question that the tetanus bacillus is the specific cause of this malady.

"Diphtheria: There is now an apparently unanimous opinion that the Klebs-Loeffler bacillus is the specific cause of diphtheria; that the exudative sore throats accompanying scarlatina, measles, and other acute diseases, while sometimes diphtheric, are usually of different origin, though not unfrequently of no less gravity."

An interesting paper was read by Dr. Martha J. Smith, of Indianapolis, on the "Effects of Modern Dress on the Health of Women." She said that American women were known abroad for their ill-health and devotion to dress. "The men are excellent theorizers on the subject," she said, "and probably have some good ideas of what the women should wear, but very few of them are willing to allow their wives or daughters to wear such costumes as are advocated by the dress-reformers whose ideas are drawn from the desire to improve the health of their sisters. The dress of the women of to-day, which compels the costal type of breathing, is certainly conducive to the development of many diseases, and proves both a predisposing and exciting cause of them. The experiments of Dr. Thomas J. Mayo prove beyond a doubt that the costal type of breathing is purely artificial, and the result of modern dress, which requires lacing. Chinese and Indian women have been examined, and the abdominal type of breathing was found in both. It has also been found that the costal type of breathing may be produced in a man by putting him in a corset. It is not the costal type of breathing that is injurious in itself, but the tight bands around the waist which necessitates it that have been found to produce the injurious results."

She then spoke of the various results of tight lacing, such as flabby flesh, increase of the size of the hips, shrinking of the muscles, limited respiration and movement of the muscles, and the "tight-lace" liver. The weight of clothing carried suspended from the waist was also criticised.

Other valuable papers were contributed, so that the full report of the meeting will make a large and valuable volume highly creditable to the Society.

